# Precision nutrition guided by genomic and functional assessments in postmenopause: a case report

**DOI:** 10.3389/fnut.2026.1724337

**Published:** 2026-01-26

**Authors:** Jenny Noland

**Affiliations:** University of Western States, Portland, OR, United States

**Keywords:** nutritional genomics, nutrigenomics-guided precision nutrition, postmenopause, estrogen metabolism, breast cancer, functional laboratory assessment, musculoskeletal function, gut–immune axis

## Abstract

Postmenopause is commonly associated with vasomotor symptoms, musculoskeletal discomfort, weight gain, gastrointestinal (GI) disturbances, and increased cardiometabolic and autoimmune risk. A 52-year-old woman presented with hot flashes, weight gain, joint stiffness and pain, and abdominal bloating. Genomic profiling revealed variants affecting methylation, detoxification, and metabolic regulation. Functional testing identified global sex hormone decline, impaired Phase II estrogen metabolism, vitamin D insufficiency, an atherogenic lipid profile, increased intestinal permeability, and elevated toxin burden. A phased, precision nutrition plan was implemented, combining dietary modification, targeted nutraceuticals, gut-directed therapy, detoxification support, sleep optimization, and stress management, alongside bioidentical hormone replacement therapy (BHRT). After 6 months, the patient experienced resolution of vasomotor symptoms and GI bloating, marked reduction in musculoskeletal pain and Medical Symptoms Questionnaire (MSQ) scores, improved sleep quality, and favorable lipid changes. This case demonstrates the potential of genomically informed precision nutrition, integrated with functional testing, to guide personalized interventions that enhance metabolic health, symptom resolution, and overall quality of life (QoL) in postmenopausal women.

## Introduction

Postmenopause is characterized by a decline in ovarian estrogen, progesterone, and androgens, contributing to vasomotor and genitourinary symptoms, weight gain (1), musculoskeletal discomfort (2), immune dysregulation (3), osteoporosis (4), sleep and mood disturbances, GI function changes, and increased cardiometabolic and cognitive risk. Although hormone replacement therapy remains the conventional approach for symptom relief, it often overlooks strategies to mitigate oncogenic risk. Genetic variability, environmental exposures, and lifestyle factors further influence hormone metabolism and therapeutic outcomes, reinforcing the need for individualized, systems-based interventions.

Precision nutrition, integrating genomic insights with functional laboratory data, provides a systems-based framework for individualized care and long-term disease prevention. Previous case reports have demonstrated that genomic profiling elucidates susceptibilities within detoxification, methylation, and inflammatory pathways (5, 6), while functional testing measures real-time endocrine, metabolic, and immune biomarkers. Together, these modalities translate genetic predispositions into actionable medical and nutritional strategies, enhancing therapeutic precision and supporting both symptom resolution and long-term health optimization (7, 8).

Despite its growing potential, clinical evidence supporting precision nutrition in menopause care remains limited. This case contributes to the emerging literature by demonstrating how genomic analysis and functional laboratory testing can inform a systems-based nutrition and lifestyle approach in a postmenopausal woman with autoimmune susceptibility, hormone imbalance, and early metabolic dysfunction (9). The findings highlight the clinical value of integrating nutrigenomics with functional medicine and nutrition to achieve symptom resolution and promote long-term health optimization.

## Case description

The patient was a 52-year-old Caucasian female working as a construction director, 17 months past her final menstrual period, presenting with hot flashes, weight gain, joint pain and stiffness, and GI bloating that had worsened during the menopausal transition. Her medical history included knee surgery, frequent childhood antibiotic exposure, and chronic ibuprofen use. The patient described adhering to a whole-food-focused diet without a defined dietary pattern, characterized by high intake of animal-based foods, limited plant diversity, and occasional alcohol consumption.

Her occupational history included chronic exposure to construction-related environmental toxins as well as sustained psychosocial stress. Family history was significant for obesity, hypertension, stroke, congestive heart failure, and GI malignancies. Despite these risks, the patient maintained an active lifestyle, high health literacy, and consistent regular stress management practices.

## Timeline of clinical care and key events

**Table:** 

Date/Follow-up	Key interventions
Pre-consult Assessment andInitial Intake (January 2025)	Diet and lifestyle assessment, nutrition-focused physical exam (NFPE), genomic and functional lab analysis (dry urine hormone testing, food allergy/sensitivity testing, toxin burden assessment, and comprehensive stool testing) Discussion on therapeutic options for vasomotor symptoms
Follow-up #1 (February 22, 2025)	Initiation of BHRT and nutraceutical regimen Elimination diet (gluten, dairy, and detected food allergens and antigens) Macronutrient adjustments (↑protein intake; ↓saturated fat intake)
Follow-up #2 (March 21, 2025)	Initiation of detox strategies (reduced toxin exposure, infrared sauna protocol) Stress management and sleep optimization
Follow-up #3 (May 21, 2025)	60-day gut cleanse protocol to reduce GI bloating and enhance gut immune function
Follow-up #4 (August 16, 2025)	Repeated blood and a dry-urine hormone test Initiation of daily flaxseed intake and resveratrol supplementation to attenuate Phase-I 4-OH estrogen metabolism Resistance training to support testosterone synthesis Synbiotic and butyrate supplements for microbial balance and mucosal integrity

## Assessment overview

Prior to the initial consultation (IC), the patient submitted recent blood work, a genomic profile, a nutrition assessment form, and an MSQ. The MSQ has been used in peer-reviewed clinical studies to assess patient-reported changes in multisystem symptoms within integrative and functional medicine research (10), supporting its utility as a practical symptom-tracking tool.

During the IC in January 2025, a detailed history review and NFPE were completed. The patient consented to additional evaluations, including a 3-day food diary; a dry urine hormone assessment (*DUTCH Complete*, Precision Analytical); stool analysis (*GI-MAP*, Diagnostic Solutions Laboratory); food allergy and sensitivity testing (*P88 Dietary Antigen Test*, Precision Point Diagnostics); and urinary toxicant screening (*Total Tox Burden*, Vibrant Wellness). These assessments were selected to establish baseline endocrine, GI, immune, and detoxification function, as they utilize widely adopted analytical platforms (e.g., LC–MS/MS for hormone metabolites, organic toxicants, and mycotoxins; ICP-MS for heavy metals; and qPCR for microbial profiling), methods that are well described in the peer-reviewed biomonitoring literature (11–13).

### Endocrine function

The DUTCH test assessment revealed global declines in sex hormones. Although Phase-I estrogen metabolism appeared balanced, reduced Phase-II methylation capacity suggested a tendency toward accumulation of reactive estrogen metabolites (8). These findings were concordant with the patient’s vasomotor symptoms and supported the implementation of a personalized bioidentical hormone replacement therapy (BHRT) regimen, with targeted methylation support informed by genomic findings (see Appendix I – Genomic Insights and Table 1: Nutraceutical regimen - Micronutrient Repletion and Methylation Support category).

**Table 1:** Nutraceutical regimen.

Category	Supplement, Daily Dosing, Ingredient Dosages Per Serving	Purposes
Micronutrient Repletion and Methylation Support	ADK Evail (*Designs for Health*): 1 softgel QD Ingredients: Vitamin D3 (5,000 IU), Vitamins A as palmitate (1515mcg), K1 + K2 (2000mcg), and Tocotrienols (50 mg)	To address low vitamin D status, support immune modulation, cardiovascular integrity, postmenopausal bone health, and antioxidant defense.
	Hydroxo B12 with Folinic Acid (*Seeking Health*): 1 cap QD Ingredients: Folinic acid (800mcg) and Hydroxocobalamin (1,000mcg) B Minus (*Seeking Health*): 1 cap QD Ingredients: P5P (20 mg), Thiamin (25 mg), Riboflavin (20 mg), Niacin (50 mg), Biotin (500 mcg), and Pantothenic Acid (150 mg)	To optimize methylation pathways impacted by FUT2, MTR, and MTRR SNPs for enhancing phase II estrogen detoxification, as indicated by the DUTCH Complete findings.
	Stellar C (Designs for Health): 1 cap QD Vitamin C (600 mg) with Bioflavonoids (quercetin, rutin, acerola)	To reduce oxidative stress and support immune resilience in the context of GSTM1 null and SOD2 SNPs.
	LDA Trace Mineral Complex (*Klair Labs*): 1 cap QD Ingredients: Iodine (200mcg), zinc (30 mg), selenium (200mcg), copper (2 mg), manganese (4 mg), molybdenum (250mcg), chromium (200mcg), potassium (100 mg), boron (200mcg), vanadium (150mcg), silica (100mcg)	To support detoxification enzymes, antioxidant activity, metabolic function, and structural integrity of connective tissue.
Hormonal, Inflammation, and Musculoskeletal Support	SynovX Relief (*Xymogen*): 1 softgel BID *Curcuma longa* and *Boswellia serrata* extract (1 g daily)	To reduce joint and muscular discomfort and modulate inflammatory cytokine activity.
	Magnesium glycinate (*Metabolic Maintenance*): 1 cap TID Ingredient: Magnesium glycinate (125 mg)	To reduce hot flashes, improve sleep quality, support stress resilience, promote bone health, enhance cardiometabolic protection, and alleviate joint discomfort.
	ProOmega 2000 (*Nordic Naturals*): 1 softgel BID EPA 1125 mg and DHA 875 mg	To address vasomotor symptoms, reduce inflammation, and improve lipid profile.
	FemGuard-HF (*Designs for Health*): 2 caps BID Ingredients: Taurine (1,000 mg), Spruce Lignans (72 mg), Gamma Oryzanol (60 mg), Genistein (54 mg), Vitamin E Isomers (50 mg) *as DeltaGold delta and gamma tocotrienols*	A synergistic combination of phytoestrogens (genistein, lignans, gamma-oryzanol, tocotrienols, and the amino acid taurine) designed to alleviate vasomotor symptoms and promote overall menopause comfort.
Digestive and Gut Barrier Support	Digestzyme V (*Ortho Molecular Products*): 1 cap with each meal Ingredients: Amylase, Protease, Lipase, Bromelain, Papain, Glucoamylase, Alpha-galactosidase, Peptidase, Lactase, Artichoke Leaf Extract, Gentian Root Extract, etc.	To compensate for low elastase-1 levels and reduce bloating.
	IgGI Shield (*Designs for Health*): 1 scoop mixed in 6 oz. of water on an empty stomach Ingredients: Serum-derived Bovine Immunoglobulin Concentrate (1 g) and N-acetyl-D-glucosamine (1 g)	To enhance mucosal immune function and intestinal barrier repair in the setting of low SIgA and elevated Zonulin.

### Cardiometabolic function

Blood chemistry (Appendix 2) revealed an atherogenic lipid pattern characterized by elevated LDL-C, LDL-P, and small, dense LDL, with low large HDL. Vitamin D deficiency and mild elevations in fasting insulin, HbA1c, and homocysteine were observed. Genomic variants in *FTO*, *APOA2*, and *TCF7L2* (Appendix 1) further indicated susceptibility to insulin resistance and adiposity, suggesting increased cardiometabolic risk.

### GI and immune function

Stool analysis (Appendix 4) demonstrated pancreatic insufficiency, intestinal dysbiosis with pathogenic overgrowth, increased permeability (↑Zonulin), and reduced mucosal immunity (↓Secretory IgA). Clinical findings, including abdominal bloating, scalloped tongue, and elevated MSQ-Digestive Tract scores (Appendix 5), further corroborated digestive insufficiency and barrier dysfunction.

Food reactivity testing (Appendix 4) revealed multiple antibody- and complement-mediated responses, consistent with immune-driven inflammation. A positive low-titer ANA (Appendix 2), broad food sensitivities, and reduced mucosal immunity indicated systemic immune activation and an autoimmune potential. Genetic variants in *HLA-DQ8*, *HLA-DQ7*, and *FUT2* (Appendix 1) further suggest a vulnerability to chronic immune dysregulation and inflammatory reactivity.

### Detoxification and oxidative stress

Genomic analysis revealed a *GSTM1* null genotype and variants in *GSTT1*, *GPX*, and *SOD2* (Appendix 1), suggesting reduced antioxidant defenses and detoxification capacity. Urinary toxin analysis (Appendix 4) confirmed a substantial load of mycotoxins and chemical metabolites, likely contributing to endocrine disruption and systemic oxidative stress.

### Musculoskeletal function

MSQ data (Appendix 5) reflected a significant burden of musculoskeletal symptoms, consistent with systemic inflammation, oxidative stress, and hormonal decline.

### Overall integration

The integration of genomic, biochemical, and clinical data revealed a network of interrelated dysfunction involving hormonal decline, impaired detoxification, toxicant accumulation, immune dysregulation, and cardiometabolic stress. This pattern reflects a complex, multisystem imbalance that warrants a precision-based, systems-oriented nutrition and lifestyle approach to restore metabolic resilience and optimize long-term health outcomes.

### Management of vasomotor symptoms: BHRT vs. diet and nutraceuticals

At the IC, the patient reported a prior trial of transdermal BHRT (BiEst with 1.5 mg estradiol/estriol and 75 mg progesterone) that alleviated vasomotor symptoms but induced GI bloating and irritability, leading to discontinuation. A subsequent nutraceutical formula provided minimal benefit. The patient expressed interest in reinitiating BHRT, emphasizing the need for a personalized formulation that would maximize efficacy while minimizing side effects and long-term cancer risk. The following two management strategies were reviewed and discussed in patient-centered terms:

#### BHRT

The patient’s genomic and DUTCH test findings indicated susceptibility to estrogen-related conditions in the context of BHRT. Homozygous variants in *CYP17A1* and *CYP19A1* suggest a predisposition toward increased estrone and estradiol synthesis. Functional alterations in *CYP17A1* may elevate androgen production and subsequent conversion to estrone and estradiol, while *CYP19A1* variants further enhance aromatase activity, collectively predisposing to estrogen excess (14–16).

Genotyping revealed no variants in *CYP1A1*, *CYP1B1*, or *CYP3A4*, indicating genetically intact Phase-I estrogen metabolism, consistent with DUTCH metabolite data. Despite the absence of a *COMT* variant, the DUTCH report showed attenuated methylation in Phase-II estrogen metabolism, reflected by a low 2-methoxy/2-hydroxyestrogen ratio at the 26th percentile. This pattern is biologically consistent with homozygous *FUT2*, *MTR*, and *MTRR* variants that may reduce S-adenosylmethionine (*SAM*) availability for *COMT*-mediated inactivation of catechol estrogens, thereby increasing the risk of reactive estrogen metabolite accumulation (17).

These findings suggest that the patient’s prior intolerance to combined estradiol/estriol/progesterone BHRT was likely attributable to genetically mediated alterations in estrogen metabolism, compounded by reduced methylation capacity. Accordingly, a conservative regimen of estriol and progesterone at the lowest effective doses, alongside targeted methylation and redox support, was discussed. DUTCH urinary estrogen metabolite profiling may be incorporated into ongoing monitoring to assess estrogen metabolism and biochemical response during BHRT, thereby supporting individualized clinical decision-making.

#### Dietary and nutraceutical strategies

Dietary and nutraceutical interventions may alleviate postmenopausal vasomotor symptoms, though the evidence is inconsistent. Phytoestrogen-rich foods, such as soy isoflavones (18) and flaxseed lignans (19), show modest benefit. Supportive nutrients, such as magnesium, B vitamins, vitamin E, and omega-3 fatty acids, may aid estrogen modulation and vascular stability. Among botanicals, black cohosh demonstrates the most consistent efficacy (20), whereas red clover and *St. John’s Wort* (19) yield variable results. Adaptogens such as maca and ashwagandha may enhance stress resilience and QoL, while evidence for chaste tree berry in postmenopausal care remains limited.

The patient elected to consider these therapeutic options and was advised to further consult her physician for evaluation. She received a summary of her DUTCH and genomic findings related to hormone synthesis and estrogen metabolism. She was encouraged to share these results to inform risk–benefit discussions and guide ongoing care.

## Intevention phase

Following the IC, the patient began a new transdermal BHRT protocol that included daily administration of 1 mg estriol and 25 mg progesterone. A personalized, systems-based nutrition care plan was developed, integrating clinical priorities, laboratory and genomic findings, and individual preferences. The six-month phased strategy targeted metabolic, immune, hormonal, GI, and toxicant-related imbalances to enhance symptom relief and QoL and minimize long-term oncogenic risk. Progress was monitored through periodic reassessment with therapeutic adjustments as indicated.

### Follow-up #1: February 22, 2025

The patient reported complete resolution of vasomotor symptoms and described a greater sense of overall well-being with the BHRT regimen. During this session, a shared decision-making process resulted in the development of an initial action plan comprising the following:

#### Sex hormone monitoring

The patient was advised to repeat the DUTCH test in 4–6 months while on BHRT to monitor changes in sex hormone levels and evaluate estrogen metabolism.

#### Dietary interventions

- Elimination Diet: The patient was advised to eliminate gluten and dairy products based on genomic insights. Although she had no history of acute IgE-mediated reactions or clinically documented food sensitivities, a 6-month elimination diet was implemented using findings from the IgE, IgG, IgG4, and C3d reactivities identified on the food allergy-sensitivity panel (Appendix 4). IgE and IgG4 patterns were evaluated together to help differentiate foods with potential IgE-mediated reactivity from those demonstrating IgG4-associated responses that may reflect immune tolerance. Because IgG-class antibodies more commonly indicate exposure rather than clinically meaningful hypersensitivity, these markers were interpreted cautiously and used as adjunctive guidance for dietary modification aimed at lowering chronic inflammatory burden. Foods demonstrating moderate-to-high C3d reactivity were targeted for elimination due to their stronger association with complement activation. A structured food reintroduction protocol was planned after the six-month elimination phase to evaluate individual tolerance.
- Macronutrient Adjustment: Based on dietary recall, the patient’s daily energy intake (~1,900 kcal) appeared to be below estimated requirements given her age, BMI, and physical activity level, with protein intake at 0.6–0.7 g/kg/day. She was advised to increase total intake to ~2,400 kcal/day, and adopt a whole-food, nutrient-dense dietary pattern emphasizing low-glycemic, phytonutrient-rich plant foods (e.g., cruciferous vegetables, leafy greens, berries). Protein intake was recommended to increase to 1.0–1.2 g/kg to support metabolic health, satiety, and preservation of lean mass. Fat quality was advised to shift from saturated-fat sources (e.g., butter, full-fat dairy, fatty cuts of meat) toward predominantly unsaturated fats, including extra-virgin olive oil, avocados, nuts, seeds, and omega-3–rich foods. This approach was tailored to her genomic profile (homozygous *FTO*, *APOA2*, and *TCF7L2* SNPs), which confer increased susceptibility to altered lipid metabolism, adiposity, impaired satiety regulation, and cardiometabolic risk. Overall, the dietary adjustments aimed to increase plant diversity, optimize macronutrient distribution, and reduce dietary exposures that contribute to systemic inflammation.

#### Nutraceutical support

A targeted nutraceutical protocol was initiated to provide support for nutrient status, intestinal barrier function, sex hormone balance, methylation efficiency, detoxification pathways, musculoskeletal function, and immune regulation (Table 1).

### Follow-up #2: March 21, 2025

One month after the first follow-up, the patient reported a 50% reduction in joint stiffness and pain following adherence to the elimination diet and supplement regimen. She consented to the following additional interventions:

- Detoxification: The patient was advised to avoid alcohol and reduce toxin exposure by wearing a facial mask at construction sites and by replacing personal care and household products with low-toxicity alternatives using Environmental Working Group (EWG) guidelines (e.g., fragrance-free cleaning products, phthalate- and paraben-free skin care, low-VOC home products). She was also instructed to implement an infrared sauna protocol (See details in Appendix 6).
- Stress Management: Continued psychotherapy, meditation, breathwork, and music therapy to promote parasympathetic activation and immune balance.
- Sleep Optimization: Consistent sleep–wake routine and light hygiene to enhance melatonin production and circadian rhythm.

### Follow-up #3: May 21, 2025

At the three-month follow-up, the patient reported sustained resolution of vasomotor symptoms, with further improvement in joint discomfort and sleep quality; however, GI bloating persisted. Stool analysis identified *Endolimax nana*, *H. pylori*, low Secretory IgA, and elevated Zonulin, consistent with dysbiosis, impaired mucosal immunity, and increased intestinal permeability. A 60-day antimicrobial and gut mucosal support protocol was initiated (Table 2).

**Table 2:** 60-day antimicrobial and mucosal support protocol.

Therapeutic agents	Daily dosing	Functions and purposes
Biocidin Broad Spectrum Liquid Formula *(Biocidin Botanicals)*	Titrate 5–15 drops, BID	Botanical antimicrobials targeting gut infections and dysbiosis.
Artemisinin Solo by *(Researched Nutritionals)*	250 mg, BID	Antiparasitic and anti-inflammatory herb targeting *Endolimax nana*.
Liver GB + by *(Biocidin Botanicals)*	2 caps, QD, 30 min before a meal	To support liver-bile function, detoxification, and digestion.
GI Detox by *(Biocidin Botanicals)*	As needed	A binder formula to prevent Herxheimer reactions and support toxin clearance.

## Outcomes

### Follow-up #4: August 16, 2025

The patient reported full resolution of GI bloating upon completing the gut protocol. Follow-up evaluations included repeat blood chemistry, DUTCH testing during BHRT, and reassessment with the MSQ. Key findings were reviewed with the patient and are summarized below.

Blood Work (Appendix 2): A comparison of baseline and follow-up lipid panels showed a marked improvement across multiple biomarkers. Total cholesterol decreased from 227 to 195 mg/dL, LDL cholesterol from 130 to 100 mg/dL, and non-HDL cholesterol from 144 to 110 mg/dL, indicating a reduced atherogenic burden and an overall improvement in cardiometabolic status.

Apolipoprotein B, a marker of atherogenic lipoprotein particle concentration, declined from 89 to 71 mg/dL, and HDL cholesterol level remained stable (83 to 85 mg/dL), reflecting maintained cardioprotective capacity. Overall, these findings reflect a marked reduction in atherogenic burden and an improvement in overall cardiometabolic status.

DUTCH Test (Appendix 3): The summary below compares baseline and follow-up DUTCH hormone results to objectively evaluate hormonal dynamics, assess therapeutic efficacy, and guide ongoing management decisions.

#### Improvements

- Progesterone and estrogens (estrone, estradiol, estriol) increased to within postmenopausal reference ranges, corresponding with resolution of vasomotor symptoms, improved sleep, and reduced joint discomfort.
- The 2-methoxy/2-hydroxyestrone ratio percentile improved from 26 to 88%, indicating enhanced methylation efficiency and clearance of reactive estrogen metabolites.

It is important to note that the observed improvement in menopausal symptoms cannot be attributed solely to the modest rise in estrogen concentrations. Clinical response to BHRT often reflects the combined influence of hormonal shifts, improved estrogen metabolism, and concurrent lifestyle and nutritional interventions implemented throughout the therapeutic period.

#### Areas for ongoing optimization

- Follow-up DUTCH testing showed a less favorable 2-OH/4-OH ratio, indicating increased 4-hydroxylation and ongoing need for antioxidant support and modulation of Phase-I estrogen metabolism.
- Testosterone remained below the expected postmenopausal range, warranting further evaluation and consideration of targeted support.

MSQ Scores (Appendix 5): MSQ scores improved markedly from January to August 2025, decreasing from 69 to 27. The greatest improvements were observed in the joints/muscles (19 → 8), weight (12 → 3), and digestive tract (10 → 2) domains, reflecting a reduction in musculoskeletal discomfort, weight-related symptoms, and GI disturbances. These findings indicate a substantial reduction in overall symptom burden over the 6 months.

### Further interventions and ongoing monitoring

The patient received structured guidance for food reintroduction. Follow-up DUTCH and serum test results informed targeted recommendations to increase intake of cruciferous vegetables and incorporate daily freshly ground flaxseed (2 tbsp/day), along with resveratrol supplementation (Resveratrol Supreme by Designs for Health, 1 capsule/day). These interventions were selected to support modulation of the 4-hydroxyestrone pathway within Phase I estrogen metabolism.

Butyrate (Tri-Butyrin Supreme by Designs for Health, 1 soft gel once daily) and synbiotic (Seed DS-01® Daily Synbiotic, 2 capsules once daily) supplements were introduced to strengthen intestinal barrier function and restore microbiota homeostasis. Continued emphasis was placed on stress management, optimization of sleep quality, regular infrared sauna therapy, and resistance training two to three times weekly to support androgen synthesis, metabolic regulation, and overall physiologic resilience.

Follow-up blood tests, the DUTCH test, and urinary toxin testing were recommended at six-month intervals to monitor progress and guide evidence-based adjustments to interventions.

## Discussion

In alignment with emerging case studies using nutrigenomic data to guide personalized dietary and lifestyle interventions in chronic disease (6, 21), this case highlights the clinical utility of integrating genomic analysis with functional laboratory assessment to inform precision nutrition and hormone management in postmenopausal women.

Genomic profiling identified homozygous variants within steroidogenic, methylation, and antioxidant pathways, including *CYP17A1*, *CYP19A1*, *FUT2*, *MTR*, *MTRR*, *APOA2*, and *FTO,* as well as *GSTM1* (null), suggesting increased estrogen synthesis, reduced methylation efficiency, and compromised oxidative defense. These genomic susceptibilities corresponded with functional evidence of impaired estrogen detoxification (low 2-methoxy/2-OH ratio), low mucosal immunity (low secretory IgA), and elevated intestinal permeability (high Zonulin). Collectively reflecting endocrine, metabolic, and immune vulnerabilities commonly observed in postmenopausal women with persistent symptom burden.

The integration of these genomic and functional insights informed a systems-based nutrition and lifestyle plan that targets hormone metabolism, detoxification, GI integrity, and immune balance. Genomic data provided predictive risk stratification, while functional testing delivered real-time biomarkers that refined intervention priorities and guided adaptive adjustments. Clinical improvements, including resolution of vasomotor symptoms, reduced musculoskeletal discomfort, and enhanced GI function and sleep quality, demonstrate the effectiveness of this integrative, data-driven model.

### Strengths and limitations

This case underscores the translational potential of precision nutrition in bridging genomic insights with functional physiology, optimizing symptom management while addressing underlying mechanisms of imbalances. Beyond the individual level, these findings align with the broader direction of nutritional science, which increasingly emphasizes integrating personalized strategies into population health frameworks. Continued research is needed to determine how genomically informed, functionally guided nutrition interventions can be scaled responsibly to support both individualized outcomes and public health optimization (9).

As a single-case observation, these findings are not generalizable and may reflect individual variability, multi-component interventions, and limitations in the reproducibility of functional tests. The six-month follow-up restricts evaluation of long-term sustainability, particularly for hormone metabolism and autoimmune risk. Environmental exposures were not systematically tracked, and the cost of genomic and functional testing may limit accessibility and broader clinical adoption.

### Patient perspective

Menopause was a confusing and frustrating time. I no longer understood my body and struggled with worsening joint pain, muscle stiffness, and headaches. Although I had completed DNA testing, bloodwork, and BHRT, I could not interpret the results or develop a plan. Working with my nutritionist, we created a targeted diet, supplement, and lifestyle protocol guided by genomic analysis and functional testing. Each change brought steady improvement. Six months later, I’m virtually pain-and hot flashes-free and able to enjoy an active life again. Her expertise has been life-changing, and I look forward to continuing this journey toward optimal health.

## Conclusion

This case demonstrates the clinical value of integrating genomic and functional laboratory data within a precision nutrition framework for postmenopausal care. Individualized, systems-based interventions improved symptoms and biomarkers by modulating hormonal, metabolic, and immune pathways. These findings support precision nutrition as a clinically relevant approach for optimizing patient outcomes and developing scalable, genomically informed health strategies.

## Data Availability

The datasets presented in this study can be found in online repositories. The names of the repository/repositories and accession number(s) can be found in the article/Supplementary material.
