# Supplementary material for: Precision nutrition guided by genomic and functional assessments in postmenopause: a case report
Source: Front Nutr. 2026 Jan 26;13:1724337. doi: 10.3389/fnut.2026.1724337 (PMC12883388; doi:10.3389/fnut.2026.1724337)
Supplement: Supplementary file 1 [file Data_Sheet_1.pdf]

APPENDCIES

Appendix 1: Genomic Insights

Homozygous SNPs are indicated in red, and heterozygous SNPs are shown in yellow.

Function	SNPs	Clinical Implications
Estrogen Synthesis	CYP19A1 TT (rs10046)	Upregulated aromatase activity → ↑Estrogen levels → ↑Risk for estrogen-sensitive conditions such as breast cancer
Androgen Synthesis	CYP17A1 GG (rs743572)	Upregulates the conversion of pregnenolone & progesterone to androgens → ↑Estrogen levels → ↑ Cancer risk
Phase I Estrogen Metabolism	CYP1A1<br>CYP1B1<br>CYP3A4	No SNPs: Normal Phase I estrogen metabolism (if sufficient methyl donors)
Phase II Estrogen Metabolism	COMT GG (val/val)	Faster enzyme activity → Efficient breakdown of catecholamines & estrogens
Methylation Insufficiency	FUT2<br>MTR<br>MTRR	FUT2 SNP: ↓B12 absorption & B12-dependent enzyme function MTR/MTRR SNPs: ↓SAMe availability - the primary methyl donor required by COMT → Potentially sluggish estrogen metabolism & ↑buildup of reactive estrogen intermediates
Metabolic Risk	FTO<br>APOA2<br>TCF7L2	FTO SNP: ↑Obesity risk, particularly via effects on appetite regulation, satiety, & fat storage.<br>APOA2 SNP: ↑Obesity & cardiometabolic risk, particularly in response to high saturated fat intake.<br>TCF7L2 SNP: ↑T2D risk, primarily through effects on insulin secretion & glucose metabolism.
Impaired Detoxification	GSTM1<br>GSTT1<br>GPx<br>SOD2	GSTM1 (null): Impaired detoxification & ↑Oxidative stress sensitivity<br>GSTT1 SNP: ↓Detoxification of xenobiotics & ↑Susceptibility to oxidative stress & toxin accumulation<br>GPx SNP: ↓Glutathione peroxidase activity → ↓Antioxidant defense & ↑Oxidative stress<br>SOD2 SNP: Impaired mitochondrial antioxidant activity → ↑Oxidative stress & inflammation
Immune Dysregulation	HLA-DQ8 & HLA-DQ7<br>FUT2	HLA haplotypes: Increased susceptibility to non-Celiac gluten sensitivity (NCGS), immune dysregulation, & autoimmunity.<br>FUT2 SNP: Impaired gut microbiome diversity & mucosal immunity, risk for autoimmune
Lactase Persistence	MCM6 (GG)	Increased risk for lactose intolerance**,** bloating, gas, or diarrhea with dairy consumption.

Appendix 2: Blood Work

		01/2025		07/2025		
**Marker**	**Functional Range**	**Patient's Result**	**Interpretation**	**Patient's Result**	**Interpretation**	**Comparison**
ANA Screen	Negative	Positive	-	Positive	-	-
ANA Titer	< 1:40	1:40	Positive (low titer)	1:40	Positive (low titer)	-
Rheumatoid Factor	< 14 IU/mL	<10	Negative	< 10	In range	-
Hs-CRP	< 1	0.7	Normal	0.6	Normal	Improved
Insulin (fasting)	2 - 5 µIU/mL	6	Above optimal	4.6		Improved
Hemoglobin A1C	4.8 - 5.2%	5.3%	Above optimal	5.1	Optimal	Improved
Homocysteine	6 – 8.5 umol/L	8.9	Above optimal	-	-	-
25(OH)D	50 - 80 ng/mL	30 ng/mL	Low	-	-	-
Thyroid Panel	Normal			Normal		-
CMP & CBC	Normal			Normal		
**Advanced Lipoprotein Panel:**						
Total cholesterol	150 - 200 mg/dL	227	High	195	Normal	Improved
LDL cholesterol	< 100 mg/dL	130	High	100	Normal	Improved

HDL cholesterol	50 - 100 mg/dL	83	Normal	85	Normal	-
Triglycerides	50 - 150 mg/dL	58	Normal	34	Normal	-
LDL particle number	< 1000 nmol/L	1399	High	-	-	-
LDL small (small dense LDL)	< 100 nmol/L	187	High	-	-	-
LDL medium	< 200 nmol/L	260	High	-	-	-
HDL large	> 7,300 nmol/L	6583	Low	-	-	-
Non-HDL cholesterol	< 130 mg/dL	144	High	-	-	-
Apolipoprotein B	< 90 mg/dL	89	Normal	71	Normal	Improved
LDL Pattern	A	A	Normal	A	Normal	-
Lipoprotein(a)	< 30 nmol/L	23	Normal	40	Normal	-

Appendix 3: DUTCH test results comparison before and after the use of BHRT

**02/2025**<br>**(Baseline before BHRT initiation)**	**08/2025**<br>**(6 months after BHRT initiation)**	**Comparison**
**Estrogens and Estrogen Metabolites:**		
**a-Pregnanediol** (Lab Range: 15-50 ng/mg):		
18.60: low end of range	28.2: in range	Improved
**b-Pregnanediol** (postmenopausal lab range: 60 - 200 ng/mg):		
83.40 ng/mg: low end of range	94.2: in range	Improved
**Total Estrogen** (postmenopausal lab range: 4.0 - 15 ng/mg):		
5.00: low end of range	9.4: in range	Improved
**Estrone:**<br>(postmenopausal lab range: 1.0-7.0 ng/mg):		
2.05: low end of range	4.31: in range	Improved
**Estradiol** (postmenopausal lab range: 0.2 - 0.7 ng/mg):		
0.21: low end of range	0.67: in range	Improved
**Estriol** (postmenopausal lab range: 0.6-4.0 ng/mg):		
1.00: low end of range	2.5: in range	Improved
**2-Methoxy/2-OH Ratio** (lab range: 0.39 - 0.67):		
0.41 (26%): low end of range	0.78 (88%): In range	Improved
**2-OH/4-OH E1 Balance** (lab range: 5.4 – 12.62):		
9.17: in range	3.90: below range	Worsened
**Androgens and Androgen Metabolites:**		
**Total DHEA** (postmenopausal lab range: 500 - 3000):		
711: low end of range	876: In range	Improved
**DHEA-S** (postmenopausal lab range: 20 - 750 ng/mg):		
25.40: low end of range	84.20: In range	Improved
**Etiocholanolone** (postmenopausal lab range: 200 – 1000 ng/mg)		
294.70: low end of range	424.10: in range	Improved
**Testosterone** (postmenopausal lab range: 2.3 – 14 ng/mg):		
2.36: below range	2.10: below range	Worsened
**Androsterone** (postmenopausal lab range: 200 – 1650 ng/mg):		
390.90: low end of range	367.50: low end of range	Worsened

Appendix 4: Key Findings of Other Functional Lab Tests

Food Allergy/Sensitivity Test

IgE, IgG4, and IgG antibodies, as well as C3d immune complement, were assessed in response to 88 different food antigens. Allergy and sensitivities to the following food antigens were identified:

IgE + IgG4 (µg/mL):

- High: Cinnamon (IgE: 10.54; IgG4 <0.05)
- Moderate: Casein (IgE: 0.72; IgG4: <0.05)), Chicken (Ig4: 0.95; IgG4: <0.08)

IgG (µg/mL):

- High: Egg yolk (161.50), Casein (58.2), Cod fish (38.06), Claim (122.88), Mustard (228.82), English walnut (135.42), Pecan (108.03)
- Moderate: Cow's milk (132.78), Goat's milk (11.66), Lima Bean (20.90), Navy Bean (20.57)

C3d (µg/mL):

- Moderate: Chicken (0.47), Egg Yolk (0.73), Navy Bean (0.50), Crab (0.37)

Urinary Total Body Burden of Toxin Test:

Toxins with levels exceeding the 95th percentile of the laboratory reference range:

- Environmental toxins: N-Acetyl (3,4-Dihydroxybutyl) Cysteine
- Heavy metals: Tin
- Mycotoxins: Fumonisins B1, Gliotoxin, Mycophenolic Acid, Zearalenone

Toxins with levels between the 75th and 95th percentiles of the laboratory reference range:

- Environmental toxins: Diethyl-thiophosphate, Dimethyldithiophosphate, Glyphosate, N-acetyl phenyl cysteine, N-acetyl-S-(2-carbamoylethyl)-Cysteine, Phenyl glyoxylic Acid, Tiglylglycine
- Mycotoxins: Sterigmatocystin, Verrucarin A

Comprehensive Stool Analysis:

Marker	Result	Clinical Relevance/Implications
Endolimax nana	High	• A non-pathogenic intestinal parasite that typically does not cause symptoms but indicates fecal-oral exposure and may co-occur with other pathogens.<br>• Its presence can be relevant in assessing gut dysbiosis or overall microbial burden.
H. pylori	High	• A pathogenic bacterium associated with chronic gastritis, peptic ulcers, and increased risk for gastric cancer.<br>• Its presence may impair digestion, disrupt stomach acid balance, and contribute to nutrient malabsorption (e.g., iron, B12).
Elastase-1	Low	• Pancreatic insufficiency →Impaired digestion → Nutrient malabsorption, gas, & bloating.<br>• Clinically, low levels warrant evaluation for underlying causes (e.g., chronic pancreatitis, diabetes) and often justify pancreatic enzyme replacement therapy and nutritional support to optimize digestion and nutrient status.
Zonulin	High	• Increased intestinal permeability → Systemic inflammation, autoimmune activation, metabolic dysfunction<br>• Clinically, it calls for targeted strategies to restore gut barrier integrity through dietary interventions, microbiome support, & specific nutraceuticals.
Secretory IgA	Low	• GI mucosal immune function in the GI tract → Increased vulnerability to infections from pathogens & opportunistic microbes, reduced defense against gut dysbiosis, candida overgrowth, or chronic GI inflammation, & impaired oral tolerance and increased risk for food sensitivities, autoimmunity, and leaky gut.<br>• Clinically, low levels may signal chronic stress, immune suppression, or long-standing gut dysfunction, and warrant support for mucosal immunity through nutrients like vitamin A, zinc, and immunoglobulin-supportive botanicals.

Appendix 5: Medical Symptom Questionnaire (MSQ) Scores

Domain	Score (01/2025)	Score (08/2025)
Joints/Muscles	19	4
Weight	12	3
Digestive Tract	10	2

Skin	6	2
Nose	5	3
Energy/Activity	4	1
Head	3	2
Eyes	3	3
Mind	3	1
Emotions	3	1
Ears	1	1
Mouth/Throat	0	0
Heart	0	0
Lungs	0	0
Other	0	0
**Grand Total**	**69**	**23**

Appendix 6: Infrared Sauna Protocol

Frequency and Timing:
Sauna sessions are recommended 2–3 times per week, conducted in the morning or early afternoon, with each session lasting approximately 30–40 minutes.

Supportive Supplements:
- Toxin binder: *PectaSol® Citrus Pectin* by EcoNugenis or *G. I. Detox* by Biocidin
- Topical Glutathione by Auro Wellness
- Buoy electrolyte drops

Sauna Instructions:
- At least 2 hours after a meal and at least 1 hour before the next meal—or first thing in the morning on an empty stomach—take either 1 capsule of *G.I. Detox* or 1 packet of *PectaSol® Citrus Pectin* mixed with 8–12 oz of water, approximately 30-40 minutes before beginning the sauna session.
- Optional: Perform 10–20 minutes of light exercise before the session to help warm the body.
- Immediately before entering the sauna, apply 2 squirts of topical glutathione to the skin.
- Remain in the sauna for 30–40 minutes, adjusting duration based on tolerance.
- Set the temperature between 125–145°F, or at a level sufficient to induce a profuse sweat.

Important Things to Watch Out For:
- Keep a cup of water in the sauna and sip regularly, before thirst develops.
- Ensure the room lights remain on during the session. Avoid using the sauna in complete darkness, as this may promote excessive parasympathetic activation and increase the risk of light-headedness or fainting, particularly upon standing.
- Maintain steady, diaphragmatic (belly) breathing throughout the session.
- Remain alert for any sensations of feeling unwell or “off”; exit the sauna immediately if these occur.
- When standing or exiting the sauna, rise slowly and stay near a wall or railing to reduce the risk of dizziness or falls.

Right After Sauna:
- Add electrolyte drops to 8–12 oz of water and drink to support rehydration.
- Shower promptly after the session to rinse sweat and residues from the skin.
